# Genotyping and outcomes of presumptive second line ART failure cases switched to third line or maintained on second line ART in Mumbai, India

**DOI:** 10.1371/journal.pone.0225631

**Published:** 2019-11-21

**Authors:** Naresh Gill, Rafael Van den Bergh, Khine Wut Yee Kyaw, Chinmay Laxmeshwar, Mrinalini Das, Sarthak Rastogi, Miriam Arago Galindo, Homa Mansoor, Stobdan Kalon, Petros Isaakidis

**Affiliations:** 1 Médecins Sans Frontières, Mumbai, India; 2 Médecins Sans Frontières, Brussels, Belgium; 3 International Union Against Tuberculosis and Lung diseases, Mandalay, Myanmar; 4 Southern Africa Medical Unit (SAMU), Médecins Sans Frontières, Cape Town, South Africa; Yeshiva University Albert Einstein College of Medicine, UNITED STATES

## Abstract

**Background:**

HIV programs are increasingly confronted with failing antiretroviral therapy (ART), including second-line regimens. WHO has provided guidelines on switching to third-line ART. In a Médecins Sans Frontières clinic in Mumbai, India, receiving referred presumptive second-line ART failure cases, an evidence-based protocol consisting of viral load (VL) testing, enhanced adherence counselling (EAC) and genotype for switching was implemented.

**Objective:**

To document the outcome and genotype of presumptive second-line ART failure cases switched to third-line or maintained on second-line ART.

**Design:**

Retrospective cohort study of patients referred between January 2011 and September 2017.

**Results:**

The cases (n = 120) were complex with median 9.2 years of ART exposure, poor adherence at baseline, and exposure to multiple ART regimens other than recommended by WHO. Out of 90 evaluated cases, 39(43%) were maintained on second-line ART. Forty-nine (54%) were ever switched to third-line ART. Twelve months virological suppression was 72% in the second-line and 93% in the third-line ART cohort, while retention in care was 80% and 94% respectively. Genotyping showed 62% resistance for PIs, and 52% triple class resistance to NRTIs, NNRTIs and PIs. Resistance was noted for the new class of integrase inhibitors, and for different drugs without any documented previous exposure to the same drug.

**Conclusion:**

Adopting WHO guidelines on switching ART regimens and provision of EAC can prevent unnecessary switching/exposure to third-line ART regimens. Genotyping is urgently required in national HIV programs, which currently use only the exposure history of patients for switching to third-line ART regimens.

## Introduction

HIV infection is a global health problem. Since the beginning of the epidemic, more than 70 million people have been infected with the virus. Globally, about 36.9 million people were reported to be living with HIV and AIDS (PLHA) at the end of 2017 and 59% were on antiretroviral therapy (ART) [1]. The Sustainable Development Goals defines an ambition to end the HIV epidemic by 2030 [2]. UNAIDS has set the ambitious 90-90-90 targets, aiming for 90% testing coverage, linkage to treatment, and viral suppression by 2020 [3]. While emphasis has been placed on meeting the first two 90s, it is essential the third 90 is also acted upon simultaneously in order to end the pandemic. In order to reach the third target (90% of all people on ART achieving viral suppression), it is essential that PLHA; 1) receive appropriate treatment regimens, including second and third line ART regimens in the case of therapeutic failure, 2) are not exposed to such regimens unnecessarily, and 3) receive adherence counselling and other support throughout their treatment.

An important element in meeting this target is the World Health Organization (WHO) recommendation of integrating routine HIV viral load (VL) testing in HIV programs, to monitor the response to ART and detect treatment failure in a timely manner [4]. However, access to routine VL testing along with enhanced adherence counseling is not keeping pace with ART scale-up, mainly due to prohibitive costs and lack of availability [5–7]. Drug sensitivity testing too is not yet a standard component of care, especially in resource-limited settings, despite the considerable threat that resistance poses to the HIV control efforts that have been realized over the past years [8,9].

India is one such country struggling to upscale its HIV program. It was home to nearly 2.1 million PLHA in 2016, the third largest number of PLHA in the world after South Africa and Nigeria [10]. Under the National AIDS Control Program, India started providing free ART in 2004 and gradually scaled up its activities in terms of facilities for treatment and number of beneficiaries [11]. ART centres provided first-line ART to 997,000 (47.5% of 2.1 million) PLHA and second line ART to 15,500 PLHA. Third-line ART was rolled out in 2016 at Centers of Excellence across India and up until September 2016, 109 PLHA had been switched to third line ART [11,12]. Although under the national HIV program, the ART centers provide free ART services, large numbers of patients still receive care from private providers for various reasons, including a lack of privacy, perceived low quality of care, and long waiting times in the public system [13,14].

WHO has defined treatment failure as consistently high VL measurements (>1000 copies of RNA/ml) while receiving adherence support for a minimum duration of 3 months in between two high VL tests [15]. However, under the national program in India, the decision to switch to third-line ART for second-line ART failure is made by a clinical expert panel at the Centers of Excellence, on the basis of a single viral load measurement (the test is repeated after 1 month if the VL is between 1000–10000 copies of RNA/ml, to rule out blips), an adherence assessment, and previous ART exposure history. Additionally, testing for antiretroviral (ARV) drug resistance by genotyping is not done [16].

Cases of presumptive second-line treatment failure/ARV resistance, without clear guidance on whether a switch in regimen is truly needed, are thus becoming increasingly common [11]. This is not without risk, as a delayed switch to second and third line ART regimens increases the risk of unfavourable outcomes, cross-resistance to even unexposed antiretroviral drugs, and primary transmission of resistant HIV subtypes in the community. An unnecessary switch to third-line ART regimens increases the risk of development of resistance against this last line of defence against HIV and increases the economic burden on the national program.

In order to answer the need for VL testing, resistance testing/genotyping, and in general appropriate provision of third-line ART treatment in Mumbai, India, the medical humanitarian non-governmental organization Médecins Sans Frontières (MSF) started providing these services at its HIV clinic in 2011. To document the strengths and challenges of this program, we undertook a study to 1) describe the socio-demographic and clinical profile of presumptive second-line ART failure cases, 2) describe the one-year retention in care and viral suppression among patients maintained on second-line ART and those switched to third-line ART treatment, and 3) document the genotypic resistance profiles.

## Methods

### Study design

This was a retrospective cohort study using routinely collected program data.

### Setting

In India, Maharashtra is the second most populous state with over 112 million inhabitants. In 2016, Maharashtra had more than 329,000 PLHA, and 25% of these were in the Mumbai. The Mumbai Districts AIDS Control Society in 2015 had 75,220 PLHA registered and 46,232 (61%) were active in care [17,18]. In Mumbai, second-line ART was available at all 16 ART centres. However, the decision to start second-line ART can only be made by two ART Plus centres (King Edward Memorial Hospital and Lokmanya Tilak Municipal Hospital) and the Centre of Excellence at Sir J.J. Group of Hospitals. Third-line ART in public sector was available only at the Centre of Excellence at Sir J. J. Group of Hospitals. Numbers of patients accessing treatment through the private sector are unknown.

In Mumbai, MSF has provided free ART services since 2006, and introduced third-line ART in January 2011. Referrals of presumptive second-line ART failure cases are received from private as well as public ART centres. The case definitions and treatment protocols used in the MSF clinic are shown in Table 1.

**Table 1:** Operational definitions and treatment protocols used in MSF Clinic.

Second-line ART	ART regimen used for treatment of PLHA who failed on first-line regimen. Typically consists of a protease inhibitor (PI) (atazanavir or lopinavir boosted with ritonavir) and 2–3 nucleoside reverse transcriptase inhibitors (NRTIs) (e.g. lamivudine and tenofovir ± zidovudine).
Third-line ART regimen	Under MSF program the second generation PI darunavir boosted with ritonavir (DRV/r) and the integras e inhibitor raltegravir (RAL), together with one or more NRTIs likely to be effective on the basis of HIV genotyping results.
Treatment Failure	Defined as a VL result > 1000 copies/ml in two consecutive results in a 3 month time interval. Adherence support should be provided for a minimum duration of 3 months in between two high VL tests, before a case is considered a treatment failure.
Second-Line Cohort	Presumptive second-line failure patients, who weren’t switched after post counselling viral load.
Third-Line Cohort	Presumptive second-line ART failure patients who were ever switched to third-line ART during the study period.
Baseline Viral Load	HIV-1 viral load at the time of referral to MSF clinic.
Post Counselling Viral Load	VL measured within 3 months post completion of 3rd EAC.
Lost to follow-up (LFU)	If the patient is missing appointment for ≥3 successive months [19].
Transferred Out (TO)	Transferred to another ART centre.
Sub-Standard regimens	The ART regimens which were not in line with advised WHO guidelines [4,15,20–23]

On referral, presumptive second-line ART failure cases are evaluated by a team of doctors, nurses, counsellors and a psychiatrist, who assess the adequacy of the treatment regimen, the adherence profile, and HIV-VL. Three sessions of enhanced adherence counselling (EAC) are offered to patients with VL >1000 copies of RNA/ml over a period of 3 months. If the post-EAC VL is higher than the cut-off value of 1000 copies of RNA/ml, then genotyping is advised. If genotyping confirms resistance, then the patient is switched to an optimal third-line ART regimen. However, if the genotype report is available at the time of referral and is suggestive of resistance to second-line ART drugs, then third-line ART may be initiated immediately and continued with treatment literacy support and EAC.

### Study population

The study population comprised all PLHA referred to the MSF clinic in Mumbai for presumptive second-line ART failure, between January 2011 and September 2017. To assess the 12 month virological suppression, a subset of patients referred up until June 2016 was considered.

### Data collection and variables

The basic socio-demographic and clinical variables (ART regimens, ART duration, CD4 count, VL at referral/baseline and follow-up) were extracted from patient files and electronic database (FUCHIA version 1.7.1.1661 software, Epicentre-MSF, Paris, France) [24]. Cases were assessed for whether their ART regimens were standard in comparison with existing WHO guidelines; as well as for adherence, opportunistic infections and comorbidities. Genotyping was interpreted based on the Stanford HIV database [25].

For programmatic outcome, a censor date of 30^th^ September 2017 was used. The possible outcomes were: alive on second-line ART, alive on third-line ART, transferred out, lost to follow-up, and died.

### Analysis and statistics

Data from the FUCHIA software was exported and analysed in EpiData Analysis software (v2.2.2.185, EpiData Association, Odense, Denmark) and SPSS (Release 20, 2011; IBM Inc., Chicago, IL, USA). All categorical variables were described using frequency tables and proportions, and associations with confirmed failure status were assessed by chi^2^ test; p-value, relative risk and 95% confidence interval were reported on. A p-value<0.05 was considered statistically significant.

### Ethics

Ethics approval was obtained from the Ethics Advisory Group of The Union, Paris, France (EAG No: 40/17).The study met the criteria for *a posteriori* analysis of routinely collected clinical data and thus did not require MSF Ethics Review Board full review. It was conducted with permission of the Medical Director, Operational Centre Brussels, MSF.

## Results

### Patient characteristics

During the study period a total of 120 patients were referred for presumptive second-line ART failure. Their baseline characteristics are shown in Table 2: 73 (61%) patients belonged to the age group 16–45 years, 89 (74%) were male, 44 (37%) had been on ART for >10 years, and 67 (56%) had CD4 count<200 cells/mm^3^. For 48 (40%) cases, VL at referral was suppressed, and 62 (52%) patients were on non-standard ART regimens at the time of referral.

**Table 2:** Baseline characteristics of presumptive second line ART failure cases referred to a Médecins Sans Frontières clinic in Mumbai, India, January 2011-September 2017 (n = 120).

Baseline Characteristics	Groups	Presumptive second line ART failure cases n (%)*
Age group	≤15 years 16–45 years >45 years	8 (7) 73(61) 39 (33)
Gender	Male Female	89 (74) 31 (26)
BMI (kg/m^2^)	BMI<18 18≤BMI<25 25≤BMI	30 (25) 59 (49) 25 (21)
ART duration	<5 years 5–9 years ≥10 years	11 (9) 52 (43) 44 (37)
Opportunistic infections	Tuberculosis Systemic fungal infection Herpes Other opportunistic infections	17 (14) 9 (8) 4 (3) 4 (3)
Co-infection (baseline)	HBV HCV	3 (2) 0 (0)
Comorbidities (baseline)	Diabetes mellitus Hypertension Cardiomyopathy Cancer No comorbidities	10 (8) 3 (2) 3 (2) 4 (3) 83 (69)
Baseline CD4 (/mm^3^)	< 100 100–200 201–500 >500	43 (36) 24 (20) 41 (34) 8 (7)
Baseline Viral load (copies/ml)	<1000 1000–10,000 10,000–99,999 ≥100,000	48 (40) 15 (13) 18 (15) 35 (29)
Adherence	Poor Good	53 (44) 58 (48)
Second line regimen	Standard Non-standard	55 (46) 62 (52)
Referring Facility	Medical NGO Governmental health facility Private health facility Non-medical referral^#^	25 (21) 46 (38) 29 (24) 19 (16)

ART: antiretroviral therapy; BMI: Body Mass Index; HBV: Hepatitis B virus; HCV: Hepatitis C virus

*The total may not add up to 120 as ‘not recorded’ values are not shown

^#^Non-medical referrals include referrals through patient self-support groups and NGOs not providing medical care.

### Retention in care and virological suppression

**Fig 1:**
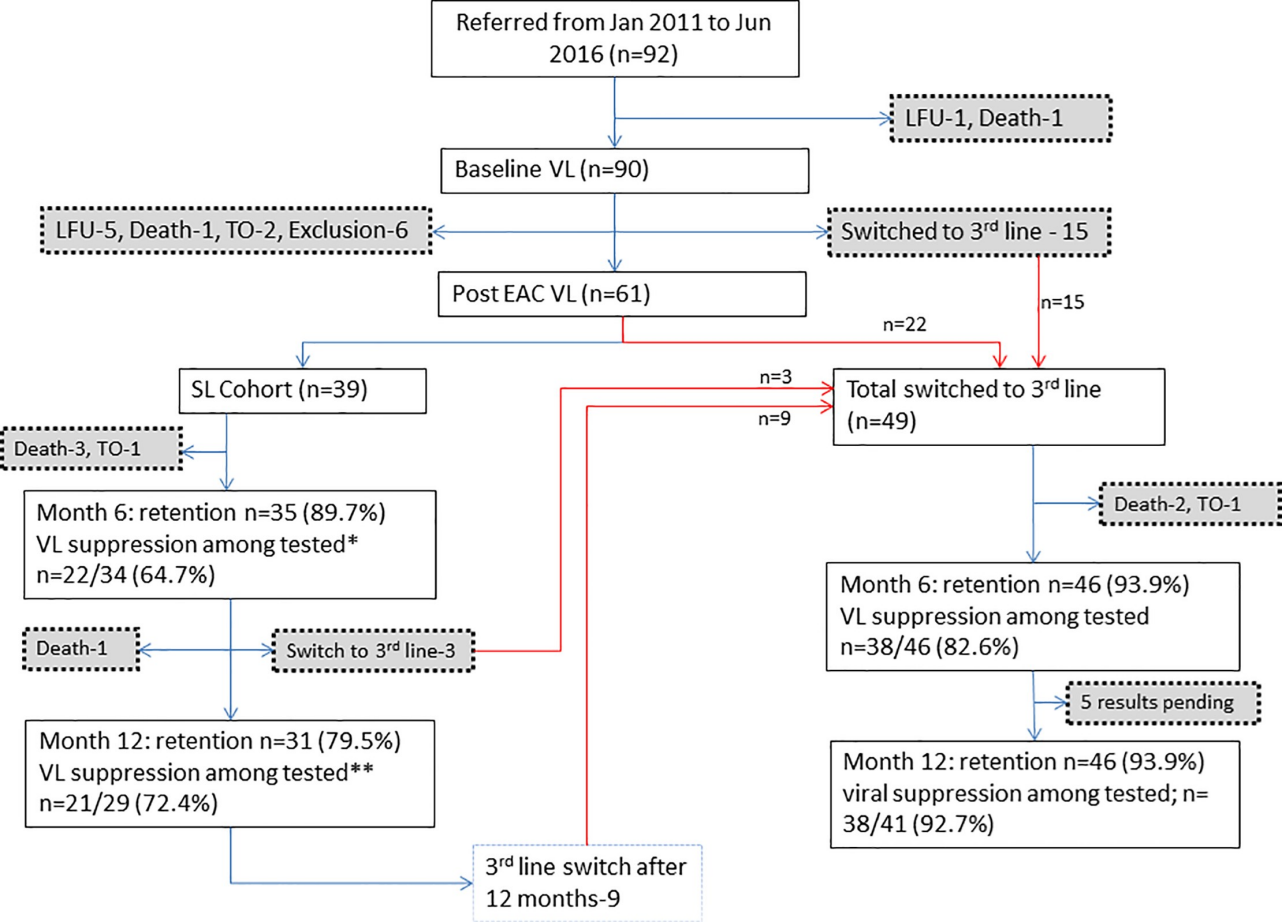
Patient flow, retention in care and viral suppression at 6 and 12 months among presumptive second line ART failure cases referred to a MSF clinic in Mumbai, India, January 2011-June 2016. * 1 SL Cohort patient were not tested at 6 months. ** 2 SL cohort patient were not tested at 12 month. SL- Second Line, LFU- Lost to Follow Up, TO- Transfer Out, EAC- Enhanced Adherence Counselling, VL- Viral Load.

In the sub-cohort of 92 patients referred till June 2016, the patient flow is described in Fig 1. Ninety patients underwent baseline VL testing. After performing the baseline VL testing, EAC, and post-counselling VL testing, 39 (43%) patients were kept on second-line ART. Among these, 6 and 12 months retention in care was 35 (90%) and 31 (79%) respectively, while virological suppression among tested was 22 of 34 (65%) and 21 of 29 (72%) respectively. Among patients switched to third-line ART at any point in time, 46 (94%) were retained both at 6 and 12 months; 38 of 46 (83%) and 38 of 41 (93%) were virologically suppressed at 6 and 12 months respectively.

### Genotyping

Among 61 patients referred with presumptive second-line ART failure who had undergone genotyping for ARV drug resistance, 10% did not show any resistance and were susceptible to all ART classes (NRTI, NNRTI and PI), while 29 (52%) showed resistance to all three ARV classes (Table 3). PI resistance was seen among 37 (62%) cases. Detailed information about mutations is provided in Table 4.

**Table 3:** Drug resistance mutation patterns among second line ART failure cases referred to a MSF clinic in Mumbai, India, January 2011-September 2017.

Drug resistance Mutation(s)		Total number tested for genotyping, n	Frequency of mutations, n (%)
Total			61
No mutation			6 (10)
At least one class detected			
	Any NRTI	61	53 (87)
	Any NNRTI	61	47 (77)
	Any PI	60	37 (62)
Mutation to 1 ARV class only			
	NRTI	56	2 (4)
	NNRTI	56	0 (0)
	PI	56	2 (4)
Mutation to 2 ARV classes			
	NRTI +NNRTI	56	17 (30)
	PI + NRTI	56	5 (9)
	PI + NNRTI	56	1 (2)
Mutation to 3 ARV classes		56	29 (52)

PI- Protease Inhibitor, NNRTI- Non nucleoside/nucleotide reverse transcriptase inhibitor, NRTI- Nucleoside reverse transcriptase inhibitor, ARV- Antiretroviral

**Table 4:** Major mutations on participants tested for drug resistance ART cases in the MSF clinic between January 2011 and September 2017.

Class	Mutations	Frequency (%)
NRTI (n = 47)	M184V	44 (93.6)
	D67N	28 (59.6)
	M41L	24 (51.1)
	K70R/E	19 (40.4)
NNRTI (n = 43)	K103N	17 (39.5)
	A98G	14 (32.6)
	K101P/H/E	12 (27.9)
	G190A	11 (25.6)
PI (n = 30)	M64I	21 (70)
	V82A/L	17 (56.7)
	I54V	12 (40)

PI- Protease Inhibitor, NNRTI- Non nucleoside/nucleotide reverse transcriptase inhibitor, NRTI- Nucleoside reverse transcriptase inhibitor, ARV- Antiretroviral

**Fig 2:**
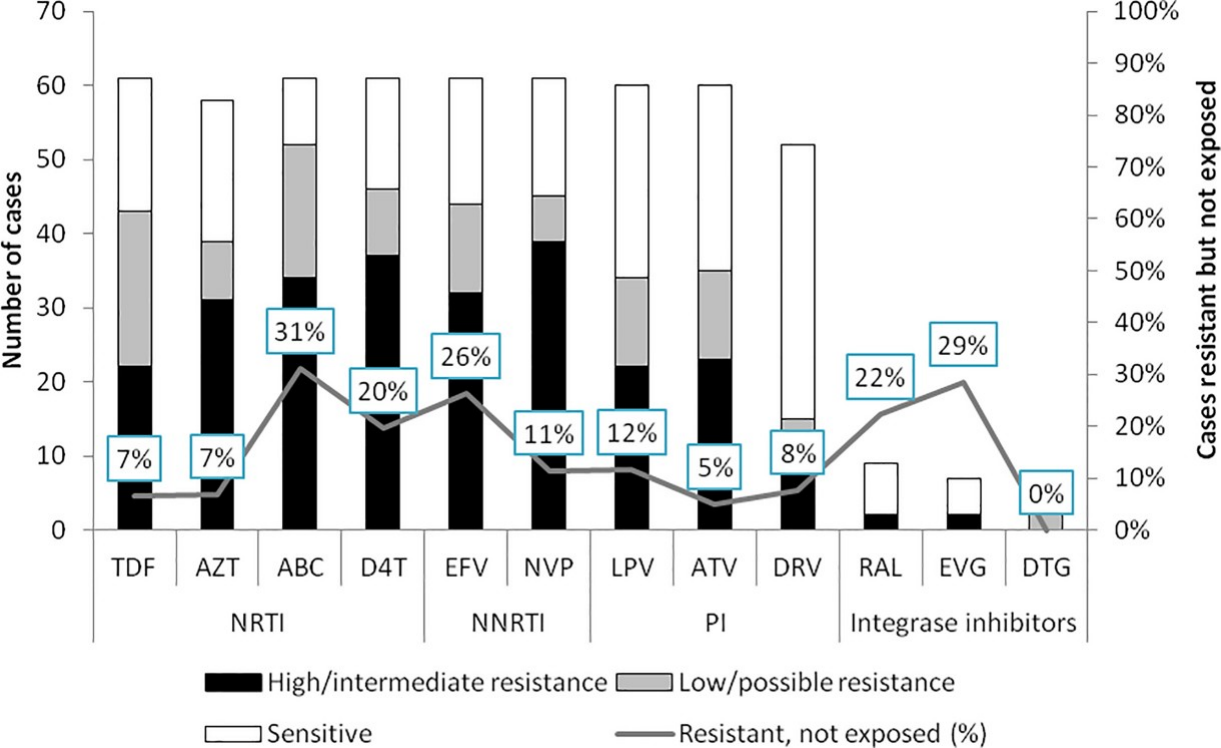
Resistance patterns in presence and absence of previous exposure to specific ARV drugs among second line ART failure cases referred to a MSF clinic in Mumbai, India, January 2011-September 2017. TDF- Tenofovir, AZT- Zidovudine, ABC-Abacavir, D4T- Stavudine, EFV (Efavirenz), NVP -Nevirapine, LPV- Lopinavir, ATV- Atazanavir, DRV-Darunavir, RAL- Raltegravir, EVG- Elvitegravir, DTG- Dolutegravir.

Intra-class cross-resistance was observed. Among NRTI drugs, resistance without previous exposure to same drug was observed among 31% of the cases for Abacavir, 20% for Stavudine, and 7% for both Tenofovir and Zidovudine; and among NNRTIs 26% for Efavirenz and 12% for Nevirapine. For PIs, 12% resistance without previous exposure to the same drug was observed for Lopinavir and 5% for Atazanavir (Fig 2).

Among the third-line drugs, resistance without previous exposure to the same drug was observed among 2/9 cases for Raltegravir and 4/52 cases for Darunavir. Of the 6 resistance tests for Dolutegravir, 2 (33%) showed low resistance without any history of exposure.

## Discussion

This study represents a cohort of presumptive second-line ART failure in adults in Mumbai treated under routine programmatic conditions. The referred cases could be considered complex, with 4 out of 10 patients having been on ART for more than 10 years; more than half on non-standard ART regimens previously, and almost half with a poor adherence at baseline. The socio-demographic and clinical profile of the patients referred to the MSF clinic for presumptive second-line ART failure was comparable to that described in the limited number of studies on similar cohorts: male predominance (60–78%), a median age of 42 years and a duration of ART exposure of 8–10 years have been documented previously [26–28].

Twelve month retention in care and viral suppression exceeded 70% among those who were maintained on second line ART and 90% in the switched group (on third line ART). Genotyping information suggested a worrying picture, with significant proportions of patients showing intra-class cross-resistance to ARVs they had not been exposed to, and with resistance observed against the integrase inhibitor class of drugs from the third-line ART regimens.

Our results support the importance of the adherence counselling in managing presumptive failure cases, as recommended by WHO [22]. Through this process, more than half of the referred cases were initially maintained on second-line ART despite the presumptive second-line failure, and more than 7 in 10 patients were still retained in care on second-line at 12 months. These indicators are comparable to existing cohort studies on second-line ART treatment [29,30], though no other studies were found to focus on a presumptive failure cohort. This is also in line with other studies documenting the retention in care after EAC in general in India and sub-Saharan Africa [26,31]. Switching to third-line ART should be based on the confirmation by consecutive high viral loads, ensuring adherence through multiple EAC sessions, and genotype results.

Third-line ART as the last line of defence against HIV was highly effective in this cohort, with over 90% retention and viral suppression at 12 months post-switching, similar to other studies [26,27]. High level of resistance against PIs, in excess of the lower levels (8–25%) documented elsewhere [31–34] but similar to those documented specifically among second-line ART failure cases (58–73%) were noted [27,28]. The triple class resistance to NRTIs, NNRTIS and PIs in more than half of the cases undergoing genotyping is worrying. In particular the presence of resistance to the new class of integrase inhibitors, albeit in a small sample size, is a major concern [35]. Finally, the existence of resistant genotypes for certain drugs even among patients without exposure to those drugs (either through cross-resistance or incomplete records of previous exposure) has major implications for the national program, as the third-line ART regimens provided through the program are designed only based on the previous exposure profile of patients. In absence of genotyping data, it seems highly likely that third-line ART regimens containing ARVs to which the patient already has resistance might be selected.

Strengths of the study include the fact that the MSF clinic provides all ART services free of cost to its beneficiaries, and hence the patient profile likely reflects that found in both the public and private health systems. Additionally, while sample sizes were relatively small and precluded a full risk factor analysis, our study represents a large cohort benefiting from genotyping and/or on third-line ART in India. A relative weakness of the study was its retrospective nature, which did not allow inclusion of key variables such as the exact start date of previous ART regimens or objective measures of adherence. Additionally, previous ARV exposures were either self-reported or were based on documents provided by patients, and it cannot be excluded that these records were incomplete. However, as the public health system also bases its design of third line regimens on self-reported or self-produced evidence of previous regimens, the implications of our observations are not affected by this study weakness.

Our study carries a number of implications. First, EAC and genotying can lead to maintaining a sizeable proportion of presumptive failure cases on second-line ART. The uptake and rigorous implementation of the EAC model is strongly encouraged.

Second, the presence of substandard regimens among more than half of all patients is indicative of the heterogeneous prescription practices that patients are exposed to over their lifelong exposure to ART. Substandard regimens compromise future treatment options for failing PLHA, especially third-line ART.

Third, the study highlights a particular urgency to make genotyping available through the national program, as documented past exposure does not seem sufficiently accurate to design an appropriate third-line regimen. Further research, possibly investigating the cost-effectiveness of genotyping is needed.

In conclusion, our study illustrates the complex challenge of providing care for presumptive second-line ART failure cases: patients with extended treatment history, substandard regimens, and poor adherence. Provision of EAC and adopting the WHO guidance on treatment switch must be encouraged. The implementation of genotyping to further guide when to switch regimens is urgently required.
